# Short-term metro passenger flow forecasting via decoupled spatio-temporal modeling with a diffusion-convergence graph and Swin-Enhanced LSTM

**DOI:** 10.1371/journal.pone.0359409

**Published:** 2026-10-06

**Authors:** Yixin Feng, Shangbing Gao, Hao Wang, Anming Bai, Gang Ren, Yuanyuan Wang, Guxue Gao, Jiahuan Ren

**Affiliations:** 1 Department of Computer and Software Engineering, Huaiyin Institute of Technology, Huaian, Jiangsu, China; 2 Trusted Firmware and Intelligent Software Laboratory, Huaian, Jiangsu, China; 3 School of Transportation, Southeast University, Nanjing, Jiangsu, China; Nanjing Forestry University, CHINA

## Abstract

Short‑term metro passenger flow forecasting—predicting station‑level inflow and outflow volumes is challenging, mainly due to the complexity of multimodal spatial couplings and the difficulty of modeling long‑range temporal dependencies while retaining local variations. Therefore, this paper proposes an Integrated Spatio-Temporal LSTM (IST-LSTM). IST-LSTM adopts a dual-branch architecture in which a diffusion branch explicitly models OD-driven propagation and a convergence branch captures structural aggregation among stations; these branches are coupled through a Diffusion and Convergence Graph (DCG) that fuses structural links, inter-station distances, and behavior-driven OD diffusion into a unified spatial representation. To strengthen temporal representation, we refine the LSTM gating mechanism by integrating a Swin Transformer module, enabling the model to better represent long-range dependencies while retaining sensitivity to local, fine-grained temporal patterns. To evaluate model generalization under dense and heterogeneous urban conditions, we construct the NJMetro dataset from Nanjing AFC smart-card transaction data, which reflects inner-ring high-density travel dynamics and pronounced spatio-temporal heterogeneity; this dataset is used alongside the public HZMetro dataset. IST-LSTM reduces RMSE by 9.19% on NJMetro relative to GCN-SBULSTM and achieves an average MAE improvement of 9.62% over Graph WaveNet on HZMetro and 8.68% versus LSTM on NJMetro. On HZMetro, it also achieves competitive RMSE performance relative to PB-GRU, with notable advantages at 15- and 30-minute horizons. Overall, the results indicate that the proposed design more effectively captures the coupled spatial and temporal mechanisms underlying metro passenger flow.

## Introduction

Urban metro systems have become indispensable components of modern urban transportation networks, offering high capacity, operational efficiency, and resilience to surface-level congestion. However, the rapid expansion of metro networks and the continuous growth in metro passenger flow have introduced new safety and management challenges, particularly under complex operating conditions and peak-hour demand surges [1]. Fluctuating passenger behaviors, heterogeneous station functions, and dynamic environmental factors often induce abrupt and highly nonlinear variations in short-term metro passenger flow. Accurately forecasting such variations is crucial for real-time traffic management, congestion mitigation, emergency response, and the efficient allocation of transport resources [2,3].

The proliferation of high-resolution mobility data and advanced sensing technologies has catalyzed a paradigm shift in intelligent transportation systems, enabling data-driven analytics across diverse applications ranging from traffic flow forecasting to automated accident reconstruction, roadside LiDAR placement optimization via high-fidelity co-simulation, and three-dimensional scene digitization [4,5]. With the increasing availability of AFC (Automatic Fare Collection) records and other high-resolution mobility data, data-driven approaches have become the dominant paradigm in traffic flow forecasting [6]. Early studies used traditional statistical and machine-learning methods (e.g., SVM, ARMA, Kalman filtering), which are interpretable and efficient but struggle with the nonlinear, non-stationary, and strongly coupled spatio-temporal dynamics of metro passenger flow [7–9]. Deep learning—especially RNNs and LSTM variants—has therefore become prevalent for modeling long-range temporal dependencies in traffic series. Nevertheless, approaches that emphasize temporal patterns alone remain inadequate when network-level spatial interactions critically shape short-term flow dynamics [10,11].

To address spatial dependencies, graph convolution techniques extend convolutional operations to non-Euclidean topologies and have given rise to a family of Graph Neural Network (GNN) models for traffic forecasting [12,13]. Representative efforts include modeling spatial relations as diffusion processes and learning adaptive adjacency matrices to capture latent node similarities [14,15]. Other works fuse physical topology, similarity, and flow correlations to enrich spatio-temporal representations. Beyond traffic-specific models, multi-scale graph interaction has emerged as a general paradigm for fusing heterogeneous relational information. Wang et al. proposed a spatial-channel collaborative multi-scale graph interaction deep transfer learning (SCMGIDTL) framework that constructs multi-scale feature prototypes and enables deep neighborhood fusion across source domains via graph-based transfer networks [16]. Despite these advances, most approaches either rely on static or single-modal spatial information or conflate different signal types in the same representation [17,18]. GNNs improve spatial modeling through graph convolutions and adaptive adjacency, but they rarely target explicit separation of propagation and station-level signals. Evidence from diffusion-based and decomposition studies suggests these perspectives are complementary, and a hybrid decoupling design could plausibly integrate their advantages [19].

Zhang et al. proposed the Spatial-Temporal Graph Diffusion Network (ST-GDN), a hierarchical GNN that models both local geographical dependencies and global inter-region diffusion while using a multi-scale attention module to capture multi-level temporal dynamics, yielding improved short-term traffic forecasting performance [20]. Jiang et al. proposed PDFormer, a propagation-delay aware long-range Transformer that models dynamic spatial dependencies and explicit time delays in inter-location propagation for improved traffic forecasting [21]. Ji and Ma proposed T-RippleGNN, a ripple-propagation attentive GNN that models topology via ripple propagation and fuses GRU temporal features with attention for improved traffic forecasting [22]. Wang et al. proposed STDGformer, which decouples temporal and spatial representations via gated transformers and then fuses them with cross-attention for improved traffic flow prediction [23].

Inspired by recent findings that separating propagation signals and local station-level signals can improve forecasts, we introduce a decoupling-based framework for short-term metro passenger-flow prediction that combines a Diffusion and Convergence Graph (DCG) to model propagation dynamics and an Integrated spatio-temporal LSTM (IST-LSTM) to capture station-level temporal patterns. The framework combines a multi-modal spatial graph that captures topology, travel-distance, and behavior-driven flow modalities with an IST-LSTM unit that embeds a Swin Transformer module into LSTM gating to strengthen temporal modeling and local contextual sensitivity [24]. To support model construction and evaluation, AFC records collected from the Nanjing rail transit authority are organized into the NJMetro dataset. Experiments on NJMetro and the public HZMetro dataset demonstrate that the proposed approach achieves near-optimal performance across evaluation metrics and improves RMSE relative to baselines on both datasets.

The main contributions of this work are summarized as follows:

(1) **Diffusion and Convergence Graph**: We propose a dual-modal convolutional feature graph that decouples OD-driven diffusion from station-level convergence signals, enabling more effective modeling of dynamic spatial dependencies in metro networks.
(2) **IST-LSTM with Swin Transformer gating:** We design a novel recurrent unit that integrates a hierarchical attention mechanism into the LSTM gate, thereby enhancing spatio-temporal feature learning and improving the capture of both long-range dependencies and fine-grained local variations.
(3) **Integrated architecture with heterogeneous attribute fusion:** We develop a dual-branch network that combines IST-LSTM and Swin Transformer modules, while simultaneously fusing structural, temporal, and behavioral attributes of metro passenger flow. This design achieves balanced modeling of global and local patterns, leading to improved robustness, predictive precision, and generalization across diverse metro scenarios.

The rest of this paper is organized as follows. Section 2 formalizes the metro passenger flow prediction problem and outlines the predictive neural network models and overall forecasting framework. Section 3 describes the construction of the newly constructed AFC dataset and presents an analysis of passenger travel characteristics. Sections 4 and 5 introduce the proposed IST-LSTM–based prediction methodology, detail the data sources, and report comparative experimental results against baseline models. Section 6 concludes the paper with a summary of key findings.

## Problem description

### Definition of metro passenger flow prediction

A metro network can be formally represented as a graph G=(V,E,A), where $V=\{V_{\text{1}},V_{\text{2}},V_{\text{3}},...,V_{n}\}$ denotes the set of stations, E⊆V×V represents the set of physical connections between stations, and A = $\left[a_{ij}\right]$ is the adjacency matrix, with $a_{ij}=1$ if stations i and j are connected, and $a_{ij}=0$ otherwise.

For each station *i*, the passenger inflow and outflow at time t are expressed as a two-dimensional vector:


$$\begin{array}{c} x_{i}^{t}=\left(x_{i,in}^{t},x_{i,out}^{t}\right) \end{array}$$ (1)

The system-wide metro passenger flow at time t is then represented as:


$$\begin{array}{c} X_{t}=(x_{\text{1}}^{t},x_{\text{2}}^{t},...,x_{n}^{t}) \end{array}$$ (2)

Given a historical observation window of length T, the short-term prediction task aims to learn a mapping function:


$$\begin{array}{c} \left(X^{t-T+1},...,X^{t})\mathrm{\backslash stackrel}\text{f}(•)\to (X^{t+1},...,X^{t+H}\right) \end{array}$$ (3)

where, H denotes the prediction horizon, f(•) is modeled as a mapping function. This formulation aligns with common practices in metro flow forecasting.

### Spatio-temporal feature extraction

Passenger flow in metro systems exhibits strong spatial correlations due to network topology and directional passenger movement patterns. Graph Convolutional Networks (GCNs) provide an effective mechanism for modeling such non-Euclidean spatial dependencies by aggregating information from neighboring stations through graph-based convolution operations [25]. This enables the extraction of spatial features that reflect both local connectivity and multi-hop propagation effects. The graph convolution operation follows the standard GCN formulation:


$$\begin{array}{c} H^{l+1}=\sigma \left({\tilde{D}}^{-\frac{\text{1}}{\text{2}}}\tilde{A}{\tilde{D}}^{-\frac{\text{1}}{\text{2}}}H^{l}W^{l}\right) \end{array}$$ (4)

where $\tilde{A}$ = *A* + *I* is the adjacency matrix with self-loops, *D̃* is the degree matrix, and $W^{l}$ is the learnable weight matrix at layer l.

Temporal dependencies, on the other hand, are typically modeled using recurrent neural networks. Long Short-Term Memory (LSTM) networks introduce memory cells and gating mechanisms that alleviate gradient vanishing and allow the model to capture long-range temporal patterns [26]. Meanwhile, Transformer architectures leverage self-attention to model global temporal dependencies and have demonstrated strong performance in sequential prediction tasks [27]. Recent studies have therefore explored hybrid architectures that integrate GCNs, LSTMs, and Transformers to jointly capture spatio-temporal correlations in traffic data.

## IST-LSTM traffic prediction model

Fig 1 presents the overall framework of IST-LSTM.

**Fig 1:**
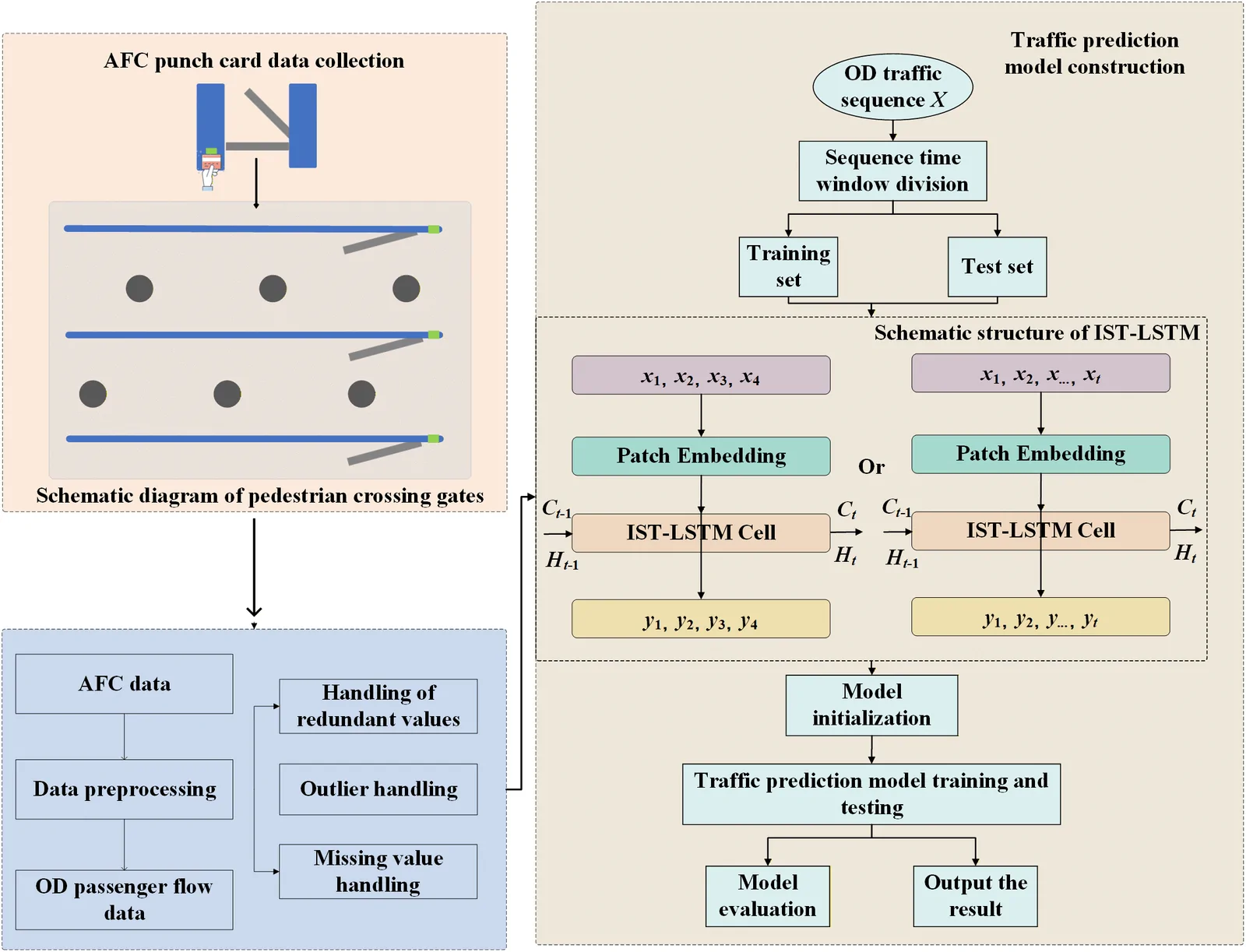
Overall framework of the proposed IST-LSTM.

### Diffusion and convergence graph (DCG) structure

Recent advances in denoising diffusion probabilistic models have demonstrated that high-fidelity signal reconstruction from uncertain, degraded observations can be effectively guided by multimodal priors and Bayesian posterior constraints [28]. The DCG draws upon this philosophy: by treating OD-driven passenger propagation as a structured diffusion process governed by multi-modal spatial priors—network topology, travel distance, and behavioral flow—the model learns to disentangle directional dispersal from station-level convergence in a manner analogous to diffusion-based restoration.

In metro networks, passenger movement is intrinsically directional and dualistic: flows originating from a given station disperse toward multiple destinations, constituting a diffusion process, whereas flows from diverse origins aggregate at a common station, constituting a convergence process. Conventional graph models typically encode spatial dependencies through symmetric or single-modal topological relationships, thereby overlooking the fundamental distinctions—both in physical mechanism and statistical property—between OD-driven directional propagation and station-level aggregation. To address this limitation, we explicitly decouple diffusion from convergence by separately modeling outbound dispersal and inbound aggregation patterns. Origin–Destination (OD) flow matrices and segment-level (inter-station) passenger counts are employed exclusively as auxiliary behavioral signals to construct the multi-modal spatial adjacency matrix within the Diffusion and Convergence Graph (DCG), thereby endowing the graph with behavior-aware spatial priors. It is emphasized that these data participate solely in the graph construction phase and do not constitute predictive targets; the prediction target remains strictly confined to station-level inflow and outflow. We subsequently construct a DCG that fuses multi-modal spatial information. The formal definitions are given as follows:

Diffusion refers to the station-level dispersal process where passenger flows from a given station propagate toward multiple destinations. Formally, for station i, the diffusion signal is defined as $\begin{array}{c} D_{i}=\sum_{j}OD_{<i,j>} \end{array}$, representing the total outflow from origin station i to all destination stations j.Convergence refers to the station-level aggregation process where metro passenger flow from multiple origins accumulate at a given station. Formally, for station j, the convergence signal is defined as $\begin{array}{c} C_{j}=\sum_{i}OD_{<i,j>} \end{array}$, representing the total inflow from all origin stations i to destination j.

The DCG as shown in Fig 2 integrates three complementary spatial modalities: (a) a multi-hop reachability matrix derived from network topology, (b) a travel-distance weight matrix computed via a Gaussian kernel on shortest path distances, and (c) a passenger-flow weight matrix constructed from OD counts and station sectional flows. Each modality is normalized and combined into a composite adjacency used by subsequent graph convolution operations. Unlike topology-only or distance-only graphs, the DCG explicitly encodes behavior-driven diffusion patterns, enabling the model to represent time-varying directional surges and asymmetric OD distributions.

**Fig 2:**
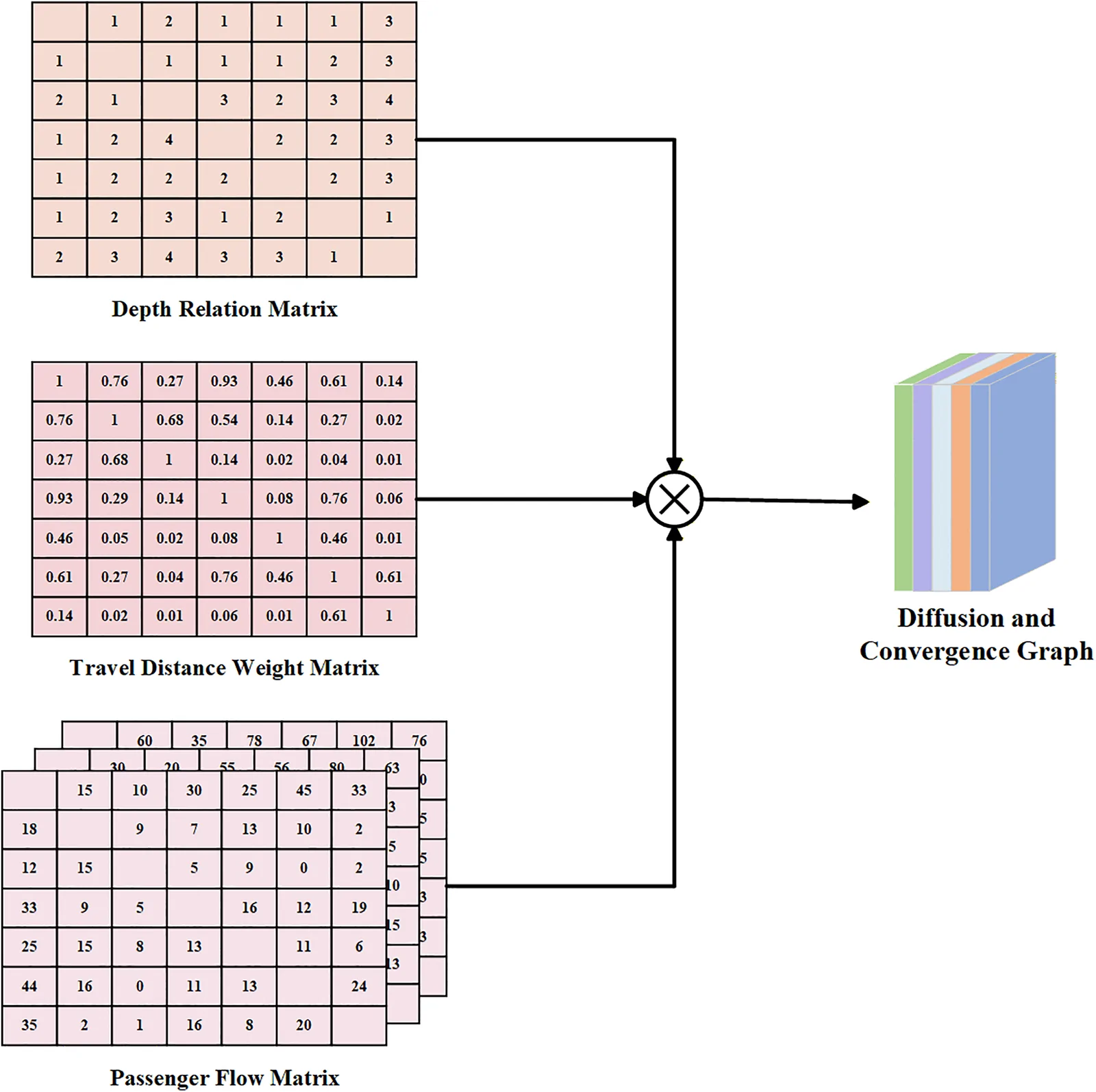
Structure of the DCG.

Formally, the DCG convergence operation integrates the three normalized modalities through a weighted combination. Let *D*, *T*, and *F* denote the multi-hop reachability matrix, travel-distance weight matrix, and passenger-flow weight matrix, respectively. The composite DCG adjacency is computed as:


$$\begin{array}{c} DCG=\alpha \cdot D+\beta \cdot T+\gamma \cdot F \end{array}$$ (5)

where α, β, γ > 0 are learnable convergence coefficients via softmax: [α, β, γ], which satisfying α + β + γ = 1. This weighted fusion constitutes the formal convergence operation that unifies structural connectivity, spatial proximity, and behavioral flow patterns into a single graph representation.

Below we detail each modality:

(1) Multi-hop Reachability Matrix: In graph convolutional networks, the symmetric normalization ${\tilde{D}}^{-\frac{\text{1}}{\text{2}}}\tilde{A}{\tilde{D}}^{-\frac{\text{1}}{\text{2}}}$ employs the inverse of the diagonal degree matrix $\tilde{D}$ to reweight neighbor contributions by node centrality. Extending this principle from single-hop degree to multi-hop topological depth, we construct a diagonal a depth connection matrix $D_{\text{1}}$ which records the structural depth of station *i* derived from a depth-first traversal of the metro network:

$$\begin{array}{c} \text{DFS(}v_{i,j}\text{)=}\left\{ \begin{array}{c} @c@\left\{v_{i,j}\right\}⋃U_{\left(v_{i,j},u\right)\in E}\text{DFS}\left(u\right),\text{if}v_{i,j}\text{is not visited}\\ \phi \text{, otherwise} \end{array}\right. \end{array}$$ (6)

where, $v_{\left(i,j\right)}$ represents the visited node, while u denotes the unvisited node. $U_{\left(v_{\left(i,j\right)},u\right)}$ signifies the edge between $v_{\left(i,j\right)}$ and the unvisited node u.

To form the Multi-hop Reachability matrix D, we add the identity matrix to obtain $D_{\text{2}}=D_{\text{1}}+I$. For a connected metro network with N stations, $D_{\text{2}}$ is strictly diagonally dominant because each diagonal entry exceeds the sum of off-diagonal entries in its row; consequently, $D_{\text{2}}$ is invertible by the Levy-Desplanques theorem. The Multi-hop Reachability matrix D is then obtained as the inverse of $D_{\text{2}}$:


$$\begin{array}{c} D=D_{\text{2}}^{-1}=\frac{\text{1}}{det\left(D_{\text{2}}\right)}\text{adj}\left(D_{\text{2}}\right) \end{array}$$ (7)

where, $D_{\text{2}}^{-1}$ denotes the inverse matrix of $D_{\text{2}}$, $det\left(D_{\text{2}}\right)$ represents the determinant of $D_{\text{2}}$, and $\text{adj}\left(D_{\text{2}}\right)$ signifies the adjoint matrix of $D_{\text{2}}$. The inverse $D_{\text{2}}^{-1}$ encodes multi-hop reachability probabilities.

(2) Travel Distance Weight Matrix: A travel distance matrix $T_{\text{0}}$ of size N × N is constructed based on the measured distances between metro stations. The Gaussian kernel function is then applied to this matrix to calculate the relative Euclidean distances between stations, forming the travel distance weight matrix T. The element $T_{i,j}$ is calculated as follows:

$$\begin{array}{c} T_{i,j}=exp\left(-\frac{\text{dist}\left(v_{i},v_{j}\right)}{2\alpha^{\text{2}}}\right) \end{array}$$ (8)

where, $\text{dist}\left(v_{i},v_{j}\right)$ indicates the shortest travel distance between stations $v_{i}$ and $v_{j}$, and α represents the standard deviation of the travel distance.

(3) Metro Passenger Flow Weight Matrix: The preprocessed dataset is incorporated into the adjacency matrix to derive the metro passenger flow matrix $F_{\text{0}}$. The sectional metro passenger flow data for each station is used as the primary feature on the diagonal, indicating the accumulated flow at each station over a period of time. The OD data is used as the global feature in the off-diagonal elements, representing the dispersed flow across stations during the same period. The metro passenger flow values in matrix F are normalized to obtain $F_{\text{1}}$. Each element’s weight is computed to yield the metro passenger flow weight matrix F, with the calculation formula for each element as follows:

$$\begin{array}{c} F_{\left(i,j\right)}=\frac{\text{1}}{\text{2}}\left(\frac{N_{\left(i,j\right)}}{\sum_{k}^{n}N_{\left(i,k\right)}}+\frac{N_{\left(j,i\right)}}{\sum_{k}^{n}N_{\left(j,k\right)}}\right) \end{array}$$ (9)

where, $N_{\left(i,j\right)}$ denotes the number of passengers traveling from station i to station j during a specific time period.

The passenger-flow weight matrix *F* inherits the directional structure of raw OD flows: each off-diagonal entry $F_{\left(i,j\right)}$ corresponds to the normalized flow from station *i* to station *j*, and no symmetrization is applied.

Unlike traditional graph constructions based only on topology or static similarity, the proposed passenger-flow weight matrix introduces a behavior-driven modality derived from real OD movements and sectional flows. This design allows DCG to capture dynamic interaction intensity—such as peak surges, directional commuting, and asymmetric distributions—beyond what topology- or distance-based matrices can represent. By bridging physical structure with actual mobility behavior, the passenger-flow matrix enhances the model’s ability to learn diffusion signals and station-level regularities, thereby improving forecasting under complex and rapidly changing metro conditions.

### Integrated spatio-temporal LSTM module

Unlike naive attention-augmented LSTMs that append a global self-attention module to the hidden state, the IST-LSTM reformulates the input gate itself as a local, hierarchical attention operator by replacing the conventional affine computation with a Swin Transformer Block (STB). At each time step *t*, the fused projection $Z_{t}\in R^{d}$ is reshaped into a 2D feature map of size (*P* × *d*/*P*), where *P* is the patch size along the temporal dimension. Non-overlapping windows of size *M* × *M* are formed, and multi-head self-attention is computed independently within each window. In alternate layers, the window partition is shifted by (*M*/2, *M*/2) to enable cross-window information exchange, allowing the model to aggregate multi-scale temporal context without quadratic complexity. Attention is applied along the temporal dimension within each station’s own feature sequence; spatial interactions are handled externally by the DCG graph convolution, keeping the STB focused on local temporal pattern extraction.

First, the current input and previous hidden state are fused via a linear projection:


$$\begin{array}{c} Z_{t}=w_{p}[x_{t};h_{t-1}]+b_{p} \end{array}$$ (10)

$$\begin{array}{c} {\hat{Z}}_{t}=\text{W-MSA}\left(\text{LN}\left(Z_{t}\right)\right)+Z_{t} \end{array}$$ (11)

$$\begin{array}{c} {Z^{\prime }}_{t}=\text{MLP}\left(\text{LN}\left({\hat{Z}}_{t}\right)\right)+{\hat{Z}}_{t} \end{array}$$ (12)

$$\begin{array}{c} {\hat{Z^{\prime }}}_{t}=\text{SW-MSA}\left(\text{LN}\left({Z^{\prime }}_{t}\right)\right)+{Z^{\prime }}_{t} \end{array}$$ (13)

$$\begin{array}{c} i_{t}=\text{MLP}\left(\text{LN}\left({\hat{Z^{\prime }}}_{t}\right)\right)+{\hat{Z^{\prime }}}_{t} \end{array}$$ (14)

where $x_{t}$ is the input feature at time *t*, $h_{t-1}$ is the previous hidden state, LN deno*t*es Layer Normalization, W-MSA and SW-MSA denote window-based and shifted-window multi-head self-attention, respectively, MLP is the feed-forward network, $i_{t}$ is the STB output.

Meanwhile, the forget gate, cell candidate, and output gate retain their standard LSTM formulations. The STB implements windowed multi-head self-attention with shifted windows, residual connections and a compact feed-forward network, providing efficient local attention and controlled cross-window exchange; this enhances responsiveness to abrupt, spatially localized flow perturbations without incurring the quadratic cost of global attention. The structure of the IST-LSTM module of IST-LSTM cell is shown in Fig 3.

**Fig 3:**
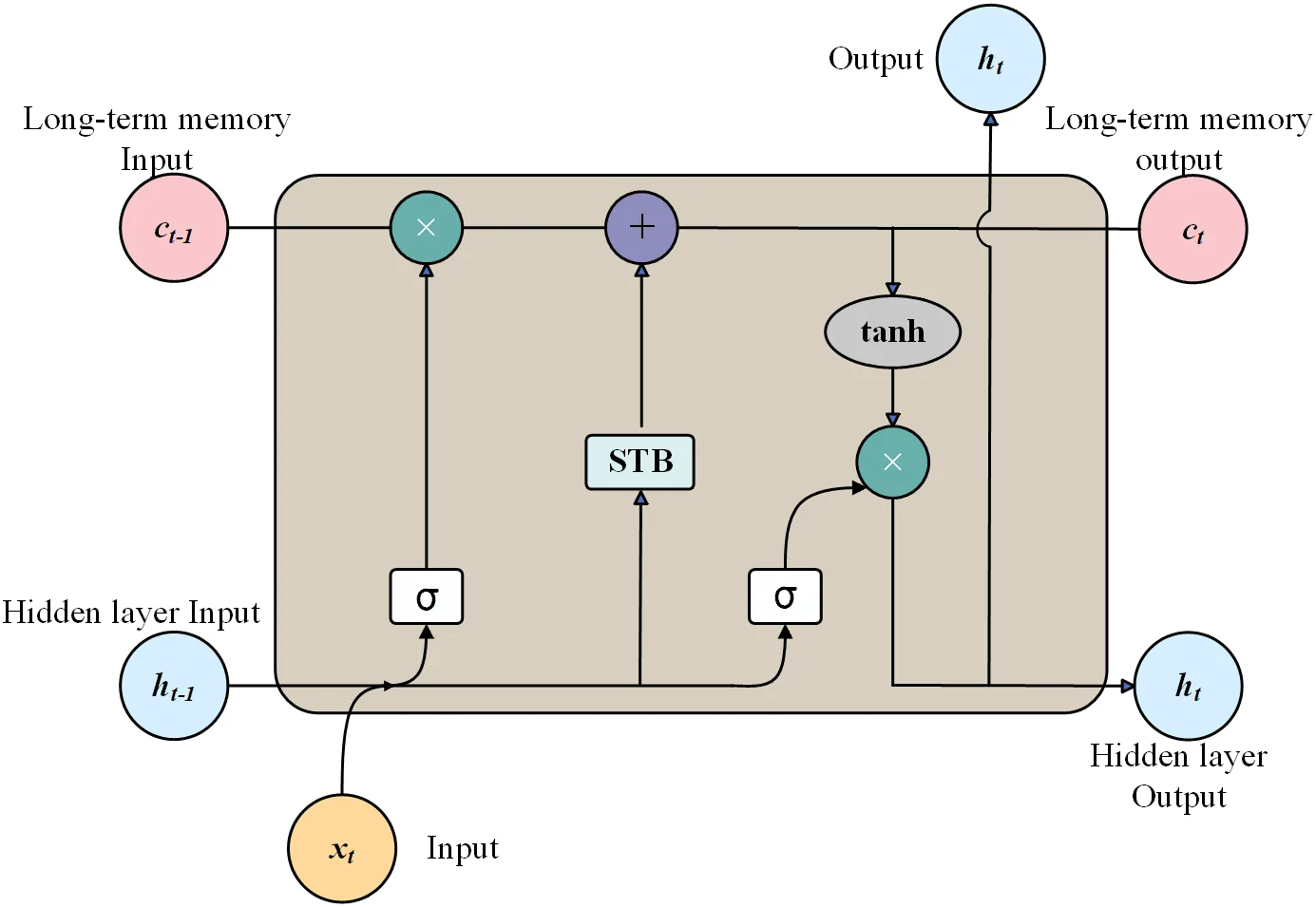
Structure of the IST-LSTM module.

### Dual‑Branch network architecture

The prediction architecture adopts a dual-branch design that explicitly models the two complementary signal types encoded in the DCG—local, station-level aggregated flows and global, OD-driven movement flows. The primary branch, implemented as a stack of three LSTM layers, focuses on extracting dominant temporal features from station-aggregated signals and thereby emphasizes stable, high-energy patterns associated with within-site accumulation and short-range diffusion. The secondary branch comprises two IST-LSTM layers and is responsible for integrating multi-scale contextual information—notably the DCG’s OD modality—and for characterizing long-range, directionally biased diffusion driven by metro passenger flow. Outputs of the primary branch are transformed and injected into the secondary branch as state activations, enabling the network to learn the dynamic interactions between local aggregation phenomena and global OD flows. This explicit decomposition, together with the state-transfer mechanism, both decouples and couples local and global signals in a principled manner, enhancing sensitivity to abrupt directional surges and asymmetric OD patterns; final forecasts are produced from the fused latent representation by a dropout-regularized fully connected layer. The overall architecture of the structure of dual‑branch architecture is illustrated in Fig 4.

**Fig 4:**
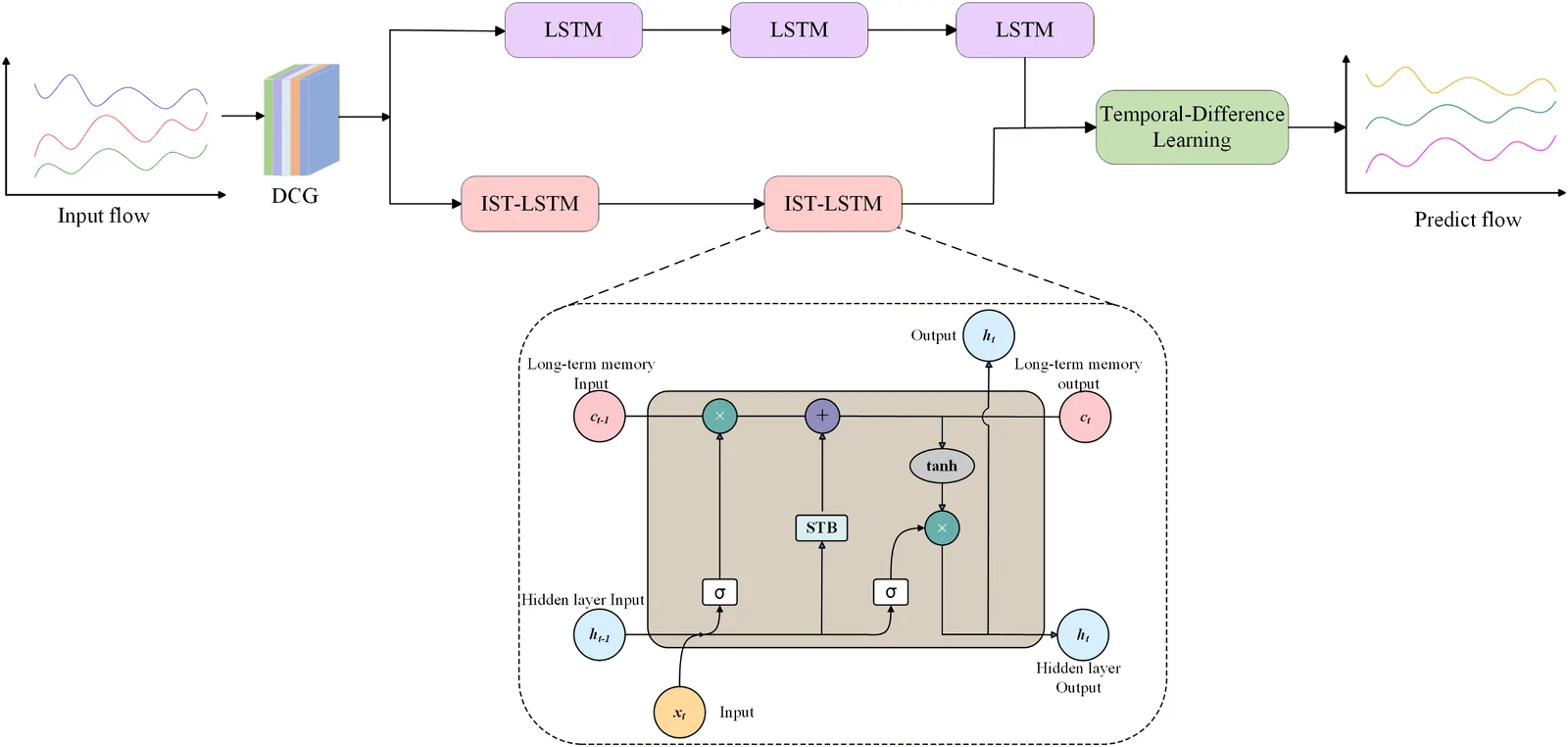
The structure of dual‑branch architecture.

The dual-branch architecture receives the station-level flow tensor $X\in R^{N\times T\times 2}$, where *N*, *T*, and 2 denote the number of stations, temporal window length, and inflow/outflow dimensions, respectively. Both branches employ the DCG adjacency applied to obtain spatial features $Z\in R^{N\times T\times d)}$ The primary branch takes Z as input and processes it through three LSTM layers, producing hidden states $H_{pri}\in R^{N\times d_{h}}$. The secondary branch concatenates raw flows with Z as $X_{sec}=[X;Z]\in R^{N\times T\times (2+d)}$ for two IST-LSTM layers, yielding $H_{sec}\in R^{N\times d_{h}}$. The IST-LSTM’s attention-enhanced gating equips the secondary branch with stronger capacity to capture OD-driven diffusion modalities.The primary state is projected and fused into the secondary branch via ${H^{\prime }}_{sec}=H_{sec}+w_{inj}\cdot H_{pri}$. Finally, $H_{pri}$ and ${H^{\prime }}_{sec}$ are concatenated and mapped by a fully-connected layer to the prediction $Y\in R^{N\times 2}$.

**Algorithm 1 Dual‑Branch network pseudocode**

**#Inputs**

X: station-level flow tensor among time T

DCG: DCG adjacency matrix

Z=GCN(DCG,X)

function_primary_branch (Z):

 $H_{p}=Z$

 for layer from 1 to 3:

$H_{p}$ =LSTM.forward($H_{p}$)

 return $H_{p}$

function_secondary_branch (Z):

 $H_{s}=DCG\left(Z\right)$

for layer from 1 to 2:

  $H_{s}$ =IST-LSTM.forward($H_{s}$)

 return $H_{s}$

**Main():**

**#Obtain the hidden states of the final layer from both branches**

$H_{pri}=function\_primary\_branch\left(Z\right)$

$H_{sec}=\text{function\_secondary\_branch}\left(Z\right)$

**# Inject the learned state into the auxiliary branch**

${H^{\prime }}_{sec}=H_{sec}+w_{inj}\cdot H_{pri}$

**# Integration and Prediction**

*fused* =Concat($H_{pri}$, ${H^{\prime }}_{sec}$)

*fused* =Dropout(fused)

Y=Linear(fused)

return Y

By replacing the input gate with a Transformer-based attention mechanism, the IST-LSTM unit can adaptively weight temporal inputs according to multi-scale contextual relevance, enabling it to capture fine-grained local variations in metro passenger flow while mitigating the gradient degradation issues associated with traditional sigmoid and tanh activations. This attention-enhanced gating also improves the model’s responsiveness to abrupt fluctuations, such as peak-hour surges or OD-driven directional shifts. Through this hybrid design, IST-LSTM functions as a powerful temporal encoder capable of jointly modeling stable inherent patterns and dynamic diffusion signals within metro passenger flow.

## Experimental results and analysis

### Data description and research area

To evaluate the effectiveness and generalization capability of the proposed model, experiments were conducted on two metro passenger flow datasets: the publicly available HZMetro dataset and a newly constructed NJMetro dataset [29]. **Table 1. Experimental dataset information.** summarizes the key characteristics of both datasets.

**Table 1:** Experimental dataset information.

dataset	HZMetro	NJMetro
Number of sites	80	159
Acquisition interval	15 minutes	5 minutes
Daily samples	72	204
Training span	19/01/1-19/01/18	21/09/1-21/09/30; 21/10/1-21/10/15
Testing span	19/01/21-19/01/25	21/10/16-21/10/27

HZMetro: The HZMetro dataset contains AFC transaction records collected from the Hangzhou metro system between January 1 and January 25, 2019. Passenger flows were aggregated at 15-minute intervals across 80 stations, yielding 72 samples per day during operating hours (05:30–23:30). Following prior studies, data from January 1–18 were used for training, and data from January 21–25 were used for testing.

**NJMetro:** The NJMetro dataset is newly constructed from AFC transaction records collected in Nanjing. It contains 159 stations and covers two non-overlapping training periods (September 1–30 and October 1–15, 2021) and one testing period (October 16–27, 2021). Raw transactions were aggregated into consistent time windows during preprocessing; feature analysis uses 5‑minute intervals while comparative experiments evaluate 15, 30, and 60‑minute forecasting granularities. Unlike HZMetro, our proprietary NJMetro dataset includes both station- and segment-level passenger-flow counts and detailed trip records, providing spatially resolved flow measurements and trip-level OD information not available in public benchmarks. The study area is confined to metro lines within Nanjing’s second-ring expressway. Compared with peripheral lines, inner-ring corridors not only exhibit the regular morning commuting peak but also sustain additional fixed demand generated by dense local service facilities, resulting in a more stable baseline ridership; citywide ridership typically falls sharply on weekends and holidays, whereas the decline is substantially smaller in the urban core. Conceptually, urban-core metro systems therefore display stronger directional commuting patterns, greater sensitivity to local events, and more pronounced diffusion-like propagation, making them representative cases for studying spatio-temporal heterogeneity. Fig 5 presents 14-day passenger-flow time series for the entire Hangzhou network (HZMetro: 2019-01-02 to 2019-01-15) and for the Nanjing inner-ring area (NJMetro: 2021-09-01 to 2021-09-14), respectively. Ethics Statement: All AFC smart‑card and passenger trip‑level mobility data used in this study were fully anonymized, with all personal identifiable information completely removed prior to analysis. The dataset was obtained under formal authorization from the local public transit authority. This research was granted an ethical exemption by the Institutional Review Board, as the processed data contained no traceable personal information.

**Fig 5:**
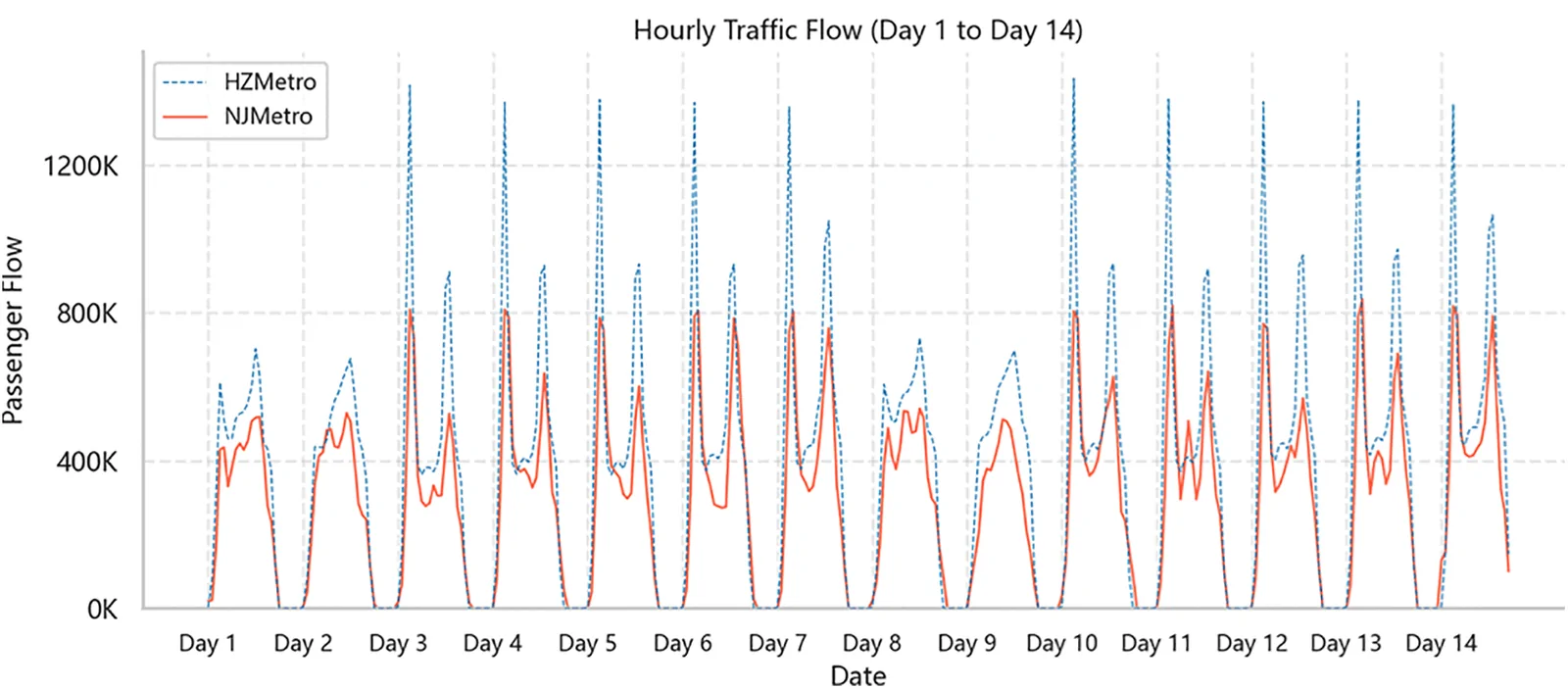
Fourteen-day passenger-flow time series for the HZMetro and NJMetro datasets.

We further analyze intraday rhythms: stable morning (07:00–09:00) and evening (17:00–20:00) peaks are observed, with the morning entry surge notably more pronounced, reflecting rigid work and school arrival times. Trip-duration statistics show 20–40 minutes accounts for 43.56% (largest share); < 20 minutes: 10.16%; > 60 minutes: 12.28%; remainder: 34.00%, indicating predominance of medium-distance commuting within the ring. During peak periods the share of short trips rises markedly (<20 minutes = 52.66%), while long trips concentrate in off-peak periods (>40 minutes = 47.34%), suggesting passengers shift travel times to avoid congestion. **The** travel time distribution of the NJMetro dataset is shown in Fig 6.

**Fig 6:**
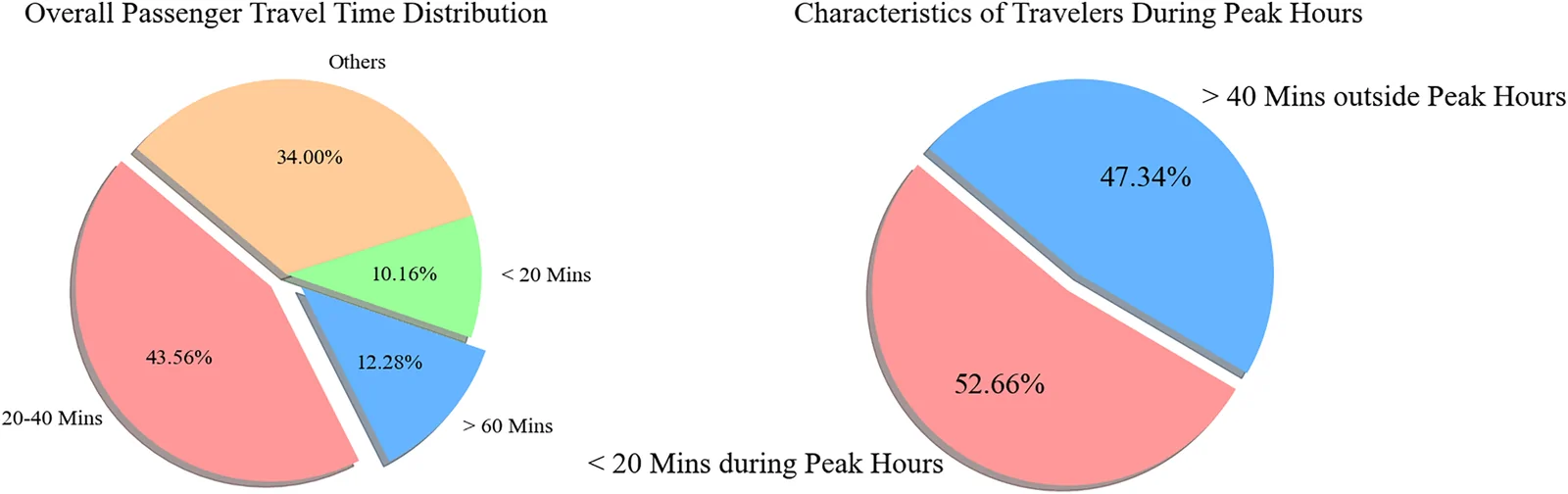
Travel time distribution of the NJMetro dataset.

### Experimental platform and training strategy

All experiments were implemented in Python 3.9.19 using the PyTorch 1.11.0 deep learning framework with CUDA 11.5 acceleration. Training was conducted on a workstation equipped with an Intel Core i9-14900K CPU, an NVIDIA RTX 4090D GPU (24 GB), and 64 GB of RAM. The model was trained using the Adam optimizer with an initial learning rate of 0.001 and a batch size of 64. The training process lasted for 200 epochs.

To ensure fair comparison, all baseline models were trained using the same data splits and optimization settings. Hyperparameters were tuned through grid search on a validation subset extracted from the training data. Mean Squared Error (MSE) was used as the loss function, while evaluation metrics included Mean Absolute Error (MAE), Root Mean Square Error (RMSE), and Mean Absolute Percentage Error (MAPE).

### Ablation study

To assess the effectiveness of the proposed IST-LSTM, we conduct three ablation experiments using the baseline LSTM model on HZMetro. Table 2 summarizes the results. Percentage improvements are computed as (Baseline − Ours)/ Baseline × 100% and averaged across the three forecasting horizons.

**Table 2:** Ablation study on model components and architecture.

model	15-minute interval			30-minute interval			60-minute interval		
	MAE	MAPE	RMSE	MAE	MAPE	RMSE	MAE	MAPE	RMSE
LSTM	23.43	14.41	40.13	24.38	15.54	42.33	26.74	19.88	47.90
${\text{LSTM}}_{\text{DCG}}$	23.01	13.89	39.71	24.17	15.34	41.98	26.51	18.69	46.01
${\text{LSTM}}_{\text{Attn}}$	22.85	13.78	39.12	24.10	15.12	41.25	26.20	18.20	45.30
${\text{I}}_{\text{st}}$	22.56	13.65	38.35	24.01	14.52	40.56	24.58	16.41	43.72
IST-LSTM	**21.89**	**13.45**	**36.01**	**22.95**	**12.98**	**39.87**	**23.75**	**14.23**	**40.52**

**Variant 1: LSTM + DCG:** It integrates only the Diffusion and Convergence Graph. Although performance slightly outperforms the baseline due to the lack of enhanced temporal modeling, DCG enriches spatial dependency encoding and provides a more expressive structural prior.

**Variant 2: LSTM + Attn Gate:** The input gate is replaced by a global multi-head self-attention module matching the STB parameter budget. This variant isolates whether gains originate from attention gating per se or from the STB’s shifted-window design. Global attention improves over the vanilla LSTM yet underperforms the STB by 0.77–1.42 in RMSE, confirming the necessity of local, multi-scale window attention for traffic periodicity.

**Variant 3: LSTM + STB Gate:** The Swin Transformer Block replaces the conventional input gate, yielding clear improvements; under the 30-minute interval, RMSE decreases by 4.2%, confirming that attention-enhanced gating captures long-range temporal dependencies and fine-grained local variations more effectively than sigmoid gates.

**Variant 4: LSTM + DCG + STB Gate:** This model combines both modules to form the full IST-LSTM, which achieves the best overall performance, reducing RMSE by 10.7% and significantly improving MAE and MAPE. This complete model also surpasses strong baselines such as GCN, Graph WaveNet, and GCN-SBULSTM, demonstrating superior capability in modeling both temporal patterns and diffusion signals.

### Experimental results and analysis

The proposed IST-LSTM model was compared with several representative baselines, including GCN, DCRNN, LSTM, SBULSTM, Graph WaveNet, and GCN-SBULSTM. Tables 3 and 4 present the quantitative results on the HZMetro and NJMetro datasets.

**Table 3:** Comparison results of different forecasting models on HZMetro dataset.

model	15-minute interval			30-minute interval			60-minute interval		
	MAE	MAPE	RMSE	MAE	MAPE	RMSE	MAE	MAPE	RMSE
GCN	24.21	21.05	49.20	25.75	24.26	52.34	33.44	27.62	62.39
LSTM	23.43	14.41	40.13	24.38	15.54	42.33	26.74	19.88	47.90
DCRNN	23.24	13.65	41.43	25.78	15.32	43.23	27.15	18.64	48.56
PVCGN [30]	22.20	**13.15**	38.12	23.13	13.87	40.01	24.55	16.35	42.26
Graph WaveNet	23.50	13.71	41.88	24.75	15.68	43.70	27.85	20.45	48.69
GCN-SBULSTM [31]	22.22	13.16	39.83	22.84	13.76	41.08	24.58	15.73	44.41
PB-GRU [32]	22.13	13.30	36.55	**22.84**	13.76	41.08	24.35	14.87	**40.02**
IST-LSTM	**21.89**	13.45	**36.01**	22.95	**12.98**	**39.87**	**23.75**	**14.23**	40.52

**Table 4:** Comparison results of different forecasting models on NJMetro dataset.

model	15-minute interval			30-minute interval			60-minute interval		
	MAE	MAPE	RMSE	MAE	MAPE	RMSE	MAE	MAPE	RMSE
GCN	25.21	21.05	50.13	24.56	26.21	52.64	34.56	28.63	61.23
LSTM	25.62	21.01	48.23	25.35	16.21	52.01	27.23	20.14	59.71
DCRNN	27.24	16.65	42.05	26.73	**16.01**	48.20	28.45	19.43	56.46
PVCGN	24.85	19.95	44.47	25.97	20.44	53.23	28.27	20.91	59.49
Graph WaveNet	23.81	20.99	46.22	27.01	20.32	53.67	32.64	25.88	65.32
GCN-SBULSTM	22.75	**16.50**	46.09	23.77	17.62	49.04	25.87	20.21	55.41
PB-GRU	23.70	17.98	42.27	23.74	17.35	47.61	26.89	20.21	51.73
IST-LSTM	**22.19**	16.78	**41.58**	**23.31**	17.81	**45.89**	**25.58**	**20.09**	**49.12**

Models that capture only a single dimension of dependency—such as LSTM (temporal only) and GCN (spatial only)—exhibit limited predictive capability. GCN performs the worst across all metrics due to its inability to model temporal evolution, while LSTM suffers from higher MAPE values, particularly at stations with low or medium passenger flow, indicating insufficient robustness to heterogeneous temporal patterns. Graph WaveNet, which integrates temporal convolution with graph convolution, performs better than GCN but still falls short of LSTM on short-interval predictions. This suggests that temporal convolution is less effective for datasets with longer sampling intervals, where long-range temporal dependencies become more prominent.

More advanced baselines such as PVCGN and GCN-SBULSTM demonstrate improved performance by incorporating multi-graph structures or bidirectional temporal modeling. However, these models rely on predefined or static adjacency matrices and do not explicitly decouple diffusion from convergence signals. As a result, they struggle to capture dynamic spatial interactions and OD-driven flow variations, especially during peak-hour surges or directional commuting flows.

In contrast, IST-LSTM achieves the best or near-best performance across the majority of metrics and forecasting horizons, with particularly strong RMSE reductions. On the HZMetro dataset, IST-LSTM achieves a 9.62% improvement in MAE over Graph WaveNet. On the NJMetro dataset, which contains more complex OD patterns and higher spatial heterogeneity, the performance gain is even more pronounced: IST-LSTM reduces RMSE by 9.19% compared with GCN-SBULSTM and achieves an 9.16% improvement in MAE relative to LSTM. These results demonstrate that the integration of DCG-based multi-modal spatial modeling and Transformer-enhanced temporal encoding enables IST-LSTM to capture both inherent temporal regularities and dynamic diffusion signals more effectively than existing methods.

Importantly, the superior performance of IST-LSTM on both the public HZMetro dataset and the newly constructed NJMetro dataset highlights its strong generalization ability and robustness. The model not only adapts well to the relatively regular passenger flow patterns in HZMetro but also performs reliably on the more complex and diverse NJMetro dataset, which features irregular OD distributions, varying station functions, and higher temporal variability. This demonstrates that IST-LSTM is capable of handling real-world metro systems with different scales, structures, and behavioral characteristics, confirming its practical applicability for short-term passenger flow forecasting. Comparison results on both datasets are presented in Tables 3–4, and Fig 7.

**Fig 7:**
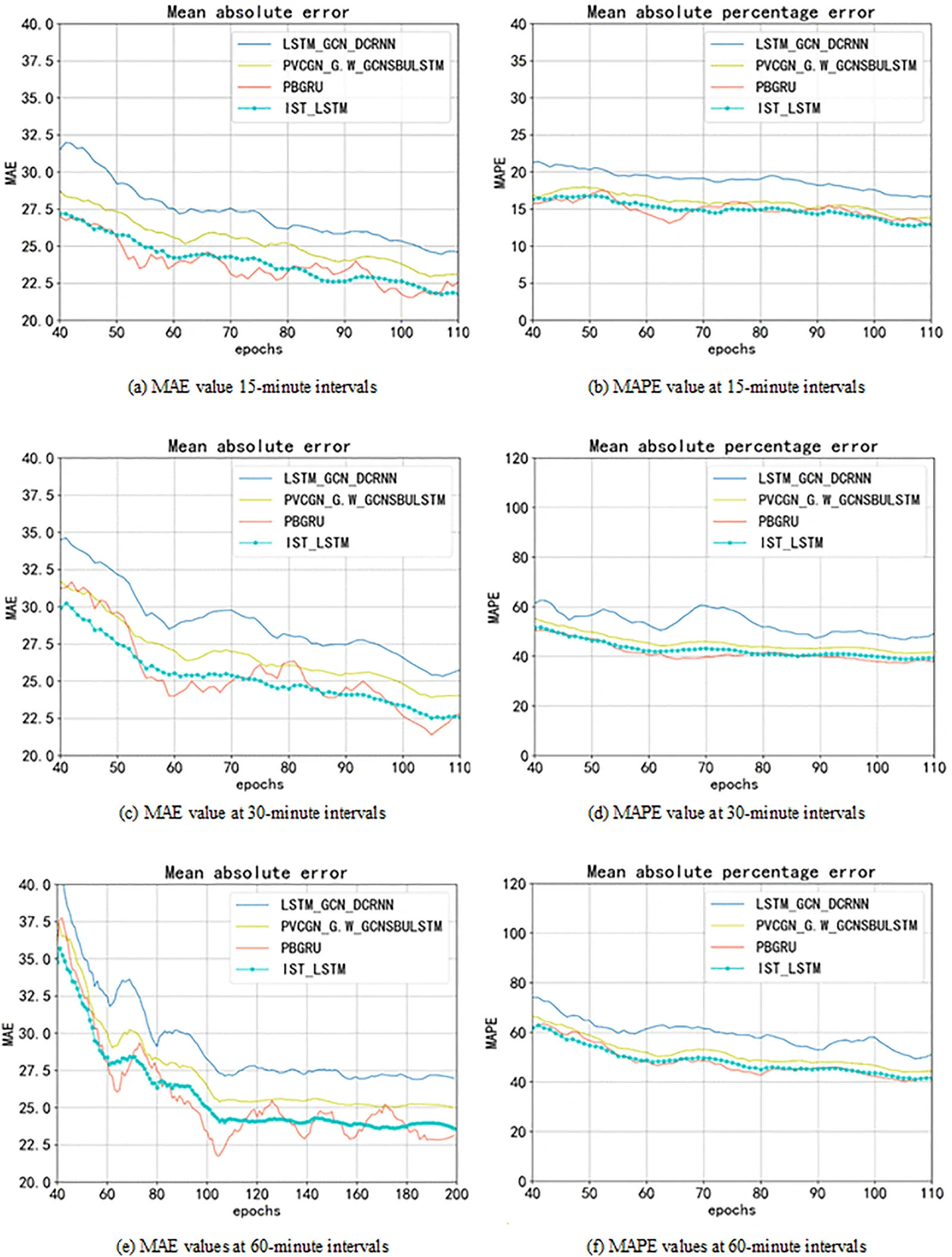
Comparison of model predictions on NJMetro dataset.

## Conclusion

This study introduces a short‑term metro passenger flow forecasting framework that integrates a multi‑modal Diffusion and Convergence Graph (DCG) with an Integrated Swin Transformer–enhanced LSTM (IST‑LSTM). By jointly modeling structural connectivity, spatial distance, and OD diffusion, DCG captures both diffusion‑like propagation and station‑level aggregation, while IST‑LSTM strengthens temporal representation through hierarchical attention. Experiments on the HZMetro and NJMetro demonstrate consistent improvements over strong baselines, with complementary gains from both components. Nevertheless, the framework entails higher computational cost, reduced robustness under irregular or event‑driven flows, and limited cross‑city validation. Future work will focus on efficiency optimization, incorporation of exogenous factors, and expansion to multi‑city datasets to further assess generalization and practical applicability.
